# Exploration of the Components and Pharmacological Mechanisms of Keyin Pill-Induced Liver Injury Based on Network Pharmacology

**DOI:** 10.1155/2022/9916949

**Published:** 2022-12-05

**Authors:** Bo Xing, Yaling Cui, Ying Chen, Lingyun Lai, Meiling Zhang, Xiangbo Xu, Nan Wang, Xiaowen Jiang, Zihua Xu, Qingchun Zhao

**Affiliations:** ^1^Department of Pharmacy, General Hospital of Northern Theater Command, Shenyang 110840, China; ^2^Department of Life Science and Biochemistry, Shenyang Pharmaceutical University, Shenyang 110016, China; ^3^Postgraduate College, China Medical University, Shenyang 110122, China

## Abstract

**Background:**

Keyin pill (KP), a patented medicine in China, is used to treat psoriasis. However, KP has been reported to have liver toxicity, but its toxic substance basis and underlying mechanisms remain unclear. Therefore, this study aimed to explore the pharmacological mechanisms and components of KP-induced liver injury through animal experiments, UPLC-QTOF/MS combined with network pharmacology.

**Methods:**

Firstly, based on the immune stress mouse model, liver function parameters and hematoxylin-eosin (H&E) staining were detected to investigate KP-induced liver injury. The UPLC-QTOF/MS method was used to identify the components of KP. CTD database and literature mining were further applied to screen nonliver protective components. Subsequently, the nonliver protective components and their corresponding targets and targets of hepatotoxicity were analyzed by the method of network pharmacology. Finally, key targets from networked pharmacology were examined by the enzyme-linked immunosorbent assay (ELISA) and molecular docking.

**Results:**

Our results indicated that KP had hepatotoxicity in male Kunming mice, which could favor hepatocyte necrosis and infiltration of inflammatory cells. A total of 70 nonliver protective compounds were identified and screened. The results of network pharmacology illustrated that methoxsalen, obacunone, limonin, and dictamnine might be the main compounds that caused liver damage. The potential hepatotoxicity mechanisms of KP might be through the IL17 and apoptosis pathways to regulate IL6, TNF*α*, CASP3, and CASP8 targets, thereby causing inflammation, excessive release of factors, and hepatocyte necrosis. The results of the ELISA experiments indicated that KP could increase the release of IL6 and TNF*α* inflammatory factors in liver tissues. Molecular docking suggested that methoxsalen, obacunone, limonin, and dictamnine had moderate binding ability with CASP3 and CASP8.

**Conclusion:**

In this study, the material basis and potential pharmacological mechanisms of KP-induced liver injury were preliminarily explored. Our research provides the initial theoretical basis for reducing the toxicity of KP.

## 1. Introduction

Keyin pill (KP) is a Chinese patent medicine composed of *Smilax glabra* Roxb*, Dictamnus dasycarpus* Turcz, the rhizome of *Menispermum dauricum*, and *Polygonum bistorta* L., which is often used in the clinical treatment of blood-heat type psoriasis [1]. Clinical studies showed that KP could alleviate psoriasis symptoms and slow down disease progression [2, 3]. However, KP could damage hepatitis and liver function in clinical application [4, 5], and the China Food and Drug Administration also issued a risk opinion for liver injury due to KP, which restricts its clinical application.

In recent years, cumulating evidence suggested the toxicity of medicinal materials in KP, including severe or even fatal liver damage, which occurred after taking *Dictamnus dasycarpus* Turcz and Chinese medicine compounds containing *Dictamnus dasycarpus* Turcz [6]; Huang et al. revealed that the obacunone, limonin, and dictamnine of *Dictamnus dasycarpus* Turcz were the main components that cause liver damage [7]; modern pharmacological studies manifested that the water extract of the rhizome of *Menispermum dauricum* could cause liver damage in mice, and the mechanism of liver damage was related to the induction of lipid peroxidation after causing oxidative stress in the body [8]; dauricine in the rhizome of *Menispermum dauricum* might be the main component of its hepatotoxicity [9]; the study prompted that *Smilax glabra* Roxb might have hepatotoxicity [10]. However, due to the complex herb composition of KP, the toxic components and mechanisms of KP leading to liver injury are still unclear. Consequently, it is necessary to carry out extensive research on the material basis and mechanisms of hepatotoxicity from KP.

In addition, KP-induced liver injury has specific properties. The incubation period of liver injury caused by KP ranged from 1 to 90 days, and the cumulative dose ranged from 20 to 1800 g. There was no difference in the dependence of liver injury on the dose and course of treatment [11]. Therefore, it is necessary to construct an animal model that can reflect KP's specificity of the liver. In the study of the toxicity of traditional Chinese medicine (TCM), the lipopolysaccharide (LPS)-induced immune stress idiosyncratic liver injury model can reflect the specificity of livers to drugs, such as the research of *Polygonum multiflorum* and Xian-Ling-Gu-Bao [12, 13].

Network pharmacology is a promising approach to investigating the comprehensive mechanisms of TCM and herbal formulae [14]. Compared with traditional toxicology, network pharmacology has the characteristics of saving resources and high speed in searching for the toxic ingredients and mechanisms of toxic Chinese herbal medicines. Thence, network pharmacology has been widely applied to explore the mechanisms of action of toxic components in TCM [15].

This study investigated the dose and pathology of KP-induced hepatotoxicity in male Kunming (KM) mice based on the immune stress model. The UPLC-QTOF/MS method was used to identify KP compounds and combine the CTD database and literature mining to screen nonliver protective components. Next, KP network analysis was performed to investigate the hepatotoxicity mechanisms and toxic ingredients of KP, including protein-protein interaction (PPI) analysis, functional enrichment analysis, and compound-target-pathway (CTP) network construction. Finally, the top-ranked targets obtained by network pharmacology were investigated by applying enzyme-linked immunosorbent assay (ELISA) experiments and molecular docking.

## 2. Methods

### 2.1. Preparation and Extraction of KP

*Smilax glabra* Roxb*, Dictamnus dasycarpus* Turcz, the rhizome of *Menispermum dauricum*, and *Polygonum bistorta* L. were purchased from Hebei Renxin Pharmaceutical Co. Ltd. (Hebei, China) and met the standards of the Chinese Pharmacopoeia (Edition 2020).

According to the preparation standard of the Ministry of Health Drug Standard Chinese Medicine Formulas Volume VI, 90 g of *Smilax glabra* Roxb, 90 g of *Dictamnus dasycarpus* Turcz, 30 g of the rhizome of *Menispermum dauricum*, and 90 g of *Polygonum bistorta* L. were mixed and decocted three times (2 hours at the first time, 1 hour at the second time, and 1 hour at the third time). The decoction was filtered and then transferred to a rotary evaporator for a concentration at 70°C to obtain the whole formula (containing 1 g of herbs per 1 mL). The whole recipe of KP was then stored in a refrigerator at 4°C.

### 2.2. Animal Experiments and Sample Collection

Animal experiments were carried out with the approval of the Ethics Committee of the General Hospital of Northern Theater Command. The experiments strictly complied with the Guide for the Care and Use of Laboratory Animals. The 18–20 g male KM mice were acquired from Liaoning Changsheng Biotechnology Co Ltd (Laboratory Animal Production License Number SCXK (Liao) 2020-0001). KM mice adapted to at least a week in the mouse room at 25°C with adequate water and food amount. The LPS (Lot No. L-2880, derived from Escherichia coli O55: B5, purified by performing phenol extraction) came from Sigma-Aldrich (MO, United States).

Based on previous studies, it was found that KP caused liver injury with special heterogeneity. Idiopathic drug-related liver injury is the body's idiosyncratic response to drugs, both allergic (immune specific) and metabolic (metabolic specific). Drug-induced liver injury induced by the organism's idiosyncratic response to a drug cannot be replicated in commonly used experimental animal models. Therefore, it is necessary to construct an animal model to reflect the specificity of the liver. In the past 30 years, studies have found that the combination of nonliver injury dose LPS and specific liver injury drugs can increase the liver injury effect of drugs on mice by inducing immune stress [16, 17]. In addition, LPS also has the toxic characteristics of targeting the liver. Therefore, in order to avoid the influence on the liver toxicity induced by KP, LPS 0.1 mg/kg without liver damage dose was selected to construct the immune stress mouse model.

**Figure 1:**
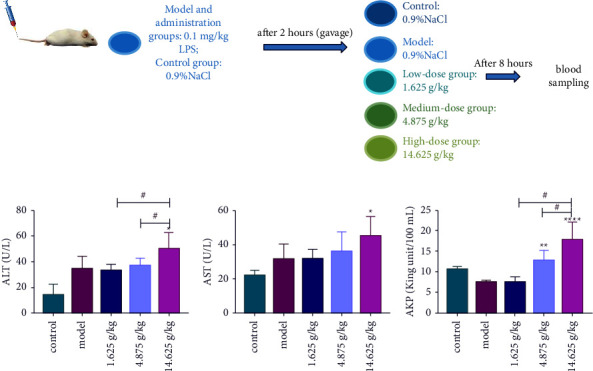
(a) Procedure of *in vivo* experiment in male KM mice. (b) Comparison of serum ALT, (c) AST, and (d) AKP levels in each group (*n* = 8 per group). Notes: ^∗^*P* value refers to the comparison between the model group and administration groups, and  ^*#*^*P* value refers to the comparison of each administration group; ^∗^*P* < 0.05, ^∗∗^*P* < 0.001, ^∗∗∗∗^*P* < 0.0001, and ^#^*P* < 0.05.

The male KM mice were randomly divided into 5 groups (*n* = 8): the high-dose group (14.625 g/kg), the medium-dose group (4.875 g/kg), the low-dose group (1.625 g/kg), the model group, and the control group. KP group and model group mice were injected with LPS 0.1 mg/kg via the tail vein. The control group was injected with 0.9% NaCl via the tail vein. After 2 hours, 14.625 g/kg (9 times the clinically equivalent dose), 4.875 g/kg (3 times the clinically equivalent dose), 1.625 g/kg (clinical equivalent dose) of KP, and 0.9% NaCl were given by gavage, respectively. After 8 hours, blood from the mice of each group was collected from the retroorbital plexus. The blood was gathered in a coagulation tube and then centrifuged at 3000 rpm in a high-speed centrifuge for 10 minutes, and then serum was collected and stored in a refrigerator at −80°C. The livers were removed and fixed in 4% paraformaldehyde and then stored at room temperature until further analysis (Figure 1(a)).

### 2.3. Serum Biochemistry

Liver function parameters, including alanine aminotransferase (ALT), aspartate aminotransferase (AST), and alkaline phosphatase (AKP) in the mouse serum were detected by using the “ALT, AST, and AKP detection kits” (Nanjing Jiancheng Institute of Biological Engineering).

### 2.4. Hematoxylin-Eosin (H&E) Staining

Liver tissues were removed from the fixed liquid. Target tissues were trimmed with a scalpel in the ventilation cupboard. Then, trimmed tissues and the label were put in the dehydration box. Next, they were dehydrated and dipped in wax. The modified tissue sections were mounted on a wax block on the paraffin microtome, and the thickness of the sections was 4 *μ*m. The sections were stained with hematoxylin solution for 3–5 minutes and rinsed with tap water. The sections were then treated with hematoxylin. Finally, the sections were stained with eosin dye for 5 minutes. Afterward, we observed through microscopic examination, image acquisition, and analysis.

### 2.5. UPLC-QTOF/MS Analysis

The processing procedure of 100 *μ*L sample of KP consisted of adding 500 *μ*L pure water, vortex mixing for 30 seconds, ultrasonic treatment with an ice bath for 1 hour, centrifugation at 12000 rpm at 4°C for 10 minutes, and removing the supernatant into a 2 mL sample bottle for machine detection. LC30A UPLC system (Waters Corp, Milford, USA) was conducted for analysis. The separation was performed on the UPLC BEH C18 column (1.7 *μ*m*∗*2.1*∗*100 mm) with gradient elution of 0.1% formic acid in liquid A water and 0.1% formic acid in liquid B acetonitrile. The sample injection volume was 5 *μ*L. Primary and secondary MS data were gathered by the AB 5600 Triple TOF TF 1.7 Mass Spectrometer Control Software Analyst, AB Sciex. In each data collection cycle, molecular ions with the strongest strength greater than 100 were selected to collect the corresponding secondary MS data. Bombardment energy: 40 eV, impact energy difference: 20 V, 15 secondary spectra every 50 ms. ESI Ion source parameters were set as follows: atomizing pressure (GS1): 55 Psi, auxiliary pressure: 55 Psi, air curtain pressure: 35 Psi, temperature: 550°C, spray voltage: 5500 V (positive ion mode) or −4000 V (negative ion mode). The raw mass spectra were imported into Progenesis QI software. The retention time correction, peak recognition, peak extraction, peak integration, peak alignment, and other work were carried out. Meanwhile, the corresponding Chinese medicine library in the compound was established, and the peaks containing MSMS data were identified by the self-builttwo-stage mass spectrometry database and the corresponding cracking law matching method.

### 2.6. Network Pharmacology

#### 2.6.1. Screening of Compounds in KP

The nonliver protective ingredients were selected as the target ingredients in combination with the literature, the Pharmacopoeia (2020 edition), and the CTD database (https://ctdbase.org/) based on the results of the UPLC-QTOF/MS analysis.

#### 2.6.2. Target Fishing of Bioactive Components and Hepatic Injury in the Database

The smiles format files for nonliver protective compounds were downloaded from the PubChem database (https://pubchem.ncbi.nlm.nih.gov/). Subsequently, smile format files were uploaded to SEA (https://sea.bkslab.org/) and Swiss Target Prediction (https://www.swisstargetprediction.ch/) databases to obtain compound-related targets. Meanwhile, the targets of nonliver protective ingredients were also screened by HERB (https://herb.ac.cn/) and CTD databases. Targets were only limited to “*Homo sapiens*” to obtain reliable results. Liver injury was used as the keyword to search for targets in GeneCards (https://www.genecards.org/) and DiGSeE (https://210.107.182.61/geneSearch/) databases. Liver injury targets with above-average relevance scores were selected from the GeneCards database, and liver injury targets of higher-than-average evidence were obtained from the DiGSeE database. Liver injury targets obtained from both databases were deduplicated and uploaded to Uniprot's website (https://www.uniprot.org/) for standardization.

#### 2.6.3. Potential Targets of Hepatotoxicity Induced by KP

The compound targets and hepatotoxicity targets were uploaded to the OmicShare online platform (https://www.omicshare.com/). The Venn function module was used to screen the overlapping targets. Intersecting targets included 556 targets associated with liver injury in the CTD database. Among the 556 targets, targets with liver injury-related scores greater than the average were screened out as direct targets of KP-induced liver injury. Direct targets of liver injury were classified by pharmacological mechanisms.

#### 2.6.4. PPI Analysis of Direct Targets

The direct targets were imported into the STRING database (https://cn.string-db.org/). The conditions were set as the PPI interaction score >0.4, and the species was restricted to “*Homo sapiens*.” The results were exported to the TSV format files. For visual analysis of direct targets, the TSV format files were imported into Cytoscape 3.7.1 software, and the “Network Analyzer” function was used to analyze the topology structure of the PPI network. Degree > mean value was set as the cut-off threshold, of which met the previously mentioned screening criteria were core targets.

#### 2.6.5. Enrichment Analysis of Gene Ontology (GO) and Kyoto Encyclopedia of Genes and Genomes (KEGG)

In order to clarify the biological functions and related signaling pathways of the targets, the screened direct targets were enriched and analyzed. GO and KEGG functional annotation of direct targets were acquired from the STRING database, with the species limited to “*Homo sapiens*.” Screening criteria were as follows: (1) false discovery rate <0.01; (2) observed gene count was greater than the average; and (3) literature has reported that signaling pathways were relevant to direct or indirect liver injury. Afterwards, the top 10 signaling pathways and GO terms that met the previously mentioned criteria were filtered out and were visualized by using the histogram and bar chart through Bioinformatics online website (https://www.bioinformatics.com.cn/).

#### 2.6.6. Construction and Analysis of the CTP Network

The corresponding targets of the top 10 signaling pathways and relevant compounds were imported into Cytoscape 3.7.1 software to construct the CTP network. The CTP network topological isomerism parameter was analyzed by the network analyzer function. The targets were regarded as key targets of liver injury induced by KP which met the following two criteria: (1) the CTP network topological isomerism parameter of degree value was greater than the mean value and (2) the topology degree value of the PPI network was greater than the average. Compounds with above-average degrees were considered potentially hepatotoxic compounds. Furthermore, combined with the structure of hepatotoxicity warning [18], the liver injury compounds of KP were screened.

### 2.7. Investigation of Key Targets

According to the results of network pharmacology, the ELISA experiments were carried out on IL6 and TNF*α* (abs520004 and abs520010, China) in liver tissues, which were the top 2 inflammatory factors.

### 2.8. Molecular Docking

In order to explore the relationship between liver injury caused by the main components of KP and apoptosis, molecular docking was applied to decipher the interaction between core compounds and hub apoptotic targets. The three-dimensional structure “SDF” files of methoxsalen, obacunone, limonin, and dictamnine were downloaded from the PubChem database. PDB format files of CASP3 (3PD1) and CASP8 (3KJQ) were downloaded from the PDB database (https://www1.rcsb.org/). Molecular docking was carried out on Maestro software (Schrödinger, USA) with the following 4 steps: protein preparation wizard, receptor grid generation, ligand preparation, and ligand docking.

### 2.9. Statistical Analysis

All data were recorded as the mean ± standard deviation. Comparisons between multiple groups were performed by using one-way ANOVA with Tukey's test using GraphPad Prism software (version 7). A value of *P* < 0.05 was considered to be statistically significant.

## 3. Results

### 3.1. Effect of KP on Serum Biochemical Markers

Liver function parameters of ALT, AST, and AKP were measured to evaluate the effect of KP on the degree of liver injury based on the immune stress model. As shown in Figures 1(b), 1(c), and 1(d), serum ALT, AST, and AKP levels were significantly increased in the high-dose group compared with the model group (*P* < 0.05). Meanwhile, compared with the model group, the serum ALT, AST, and AKP levels in the medium-dose group had a rising tendency. There was no significant difference in liver function parameters between the low-dose group and the model group. Biochemical analysis results indicated that KP had a certain degree of liver injury in KM mice when given a high dose of 14.625 g/kg. KP had potential hepatotoxicity when given a medium dose of 4.875 g/kg.

### 3.2. Histopathological Evaluation of Hepatic Tissue after Administration of KP

**Figure 2:**
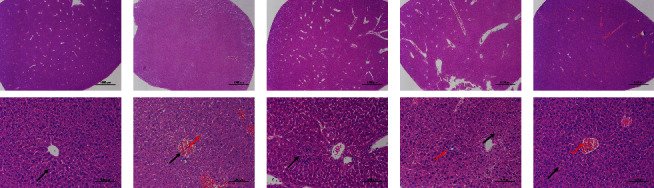
H&E staining (scale: top row: 20X, 1000* μ*m; lower row: 200X, 100 *μ*m) of liver tissue in each group. (a) control group, (b) model group, (c) low-dose of the KP group, (d) medium-dose of the KP group, and (e) high-dose of the KP group.

H&E staining was applied to observe the histopathological changes in liver tissues in each group. In the control group, there was a large amount of hepatocyte steatosis, with tiny round vacuoles in the cytoplasm (black arrow) and no obvious inflammatory changes (Figure 2(a)). Compared with the control group, the model group had a large amount of vascular congestion (black arrow), a small amount of hepatic cell steatosis around the portal area, and tiny round vacuoles in the cytoplasm (red arrow), and no obvious inflammatory changes (Figure 2(b)). Pathological results from the low-dose group showed focal necrosis of liver lobules, nuclear fragmentation, and a small amount of inflammatory cell infiltration (black arrow) (Figure 2(c)). The pathological results of the medium-dose group showed a large number of hepatic cell steatosis and tiny round vacuoles (black arrow) in the cytoplasm and had a lobule with focal necrosis of liver cells and fragmentation of nuclei with a small amount of inflammatory cell infiltration (red arrow) (Figure 2(d)). In the high-dose group, histopathological analysis of the liver revealed that there was necrosis of liver cells, fragmentation of nuclei, and massive blood vessel congestion (Figure 2(e)). H&E staining indicated that administration of KP could cause cell necrosis and inflammatory cell infiltration in the liver of mice.

### 3.3. Identification of Bioactive Ingredients from KP

**Figure 3:**
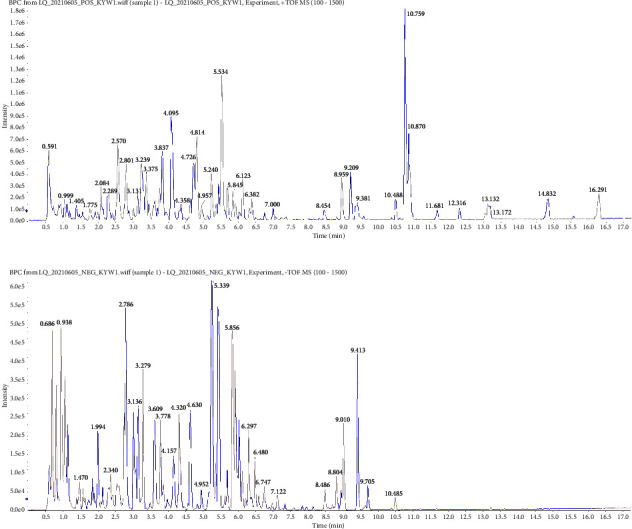
UPLC-QTOF/MS results. (a) KP with positive ion mode. (b) KP with negative ion mode.

UPLC-QTOF/MS was used to qualitatively analyze the chemical constituents of KP. The total ion chromatograms (TIC) of KP were scanned in positive and negative ion modes (Figures 3(a) and 3(b)). Based on UPLC-QTOF/MS analysis results, 70 nonliver-protective ingredients were screened by the CTD database, Pharmacopeia (2020), and literature consulting (Table 1).

### 3.4. Acquisition of Component Targets and Hepatotoxicity Targets

A total of 1618 component targets were screened from SEA, Swiss target prediction, HERB, and CTD databases after removing the duplicated targets. There were 2381 targets with an above-average relevance score that were filtered through the GeneCards database, and 106 targets with higher-than-average evidentiary results were achieved in the DiGSeE database. Subsequently, 2357 liver toxicity targets were obtained after normalization by the Uniprot database. There were 564 intersection targets that were obtained. Among the intersection targets, 556 targets were associated with liver injury in the CTD database. There were 87 targets with higher-than-average inference scores of liver injury evidence in the CTD database. Consequently, the 87 targets were considered to be direct targets of KP-induced liver injury. Direct targets were classified into 6 categories including the metabolic enzyme family, the transporter family, apoptosis, inflammation, nuclear hormone receptors, and oxidative stress according to the pharmacological mechanisms.

### 3.5. PPI Network Analysis

**Figure 4:**
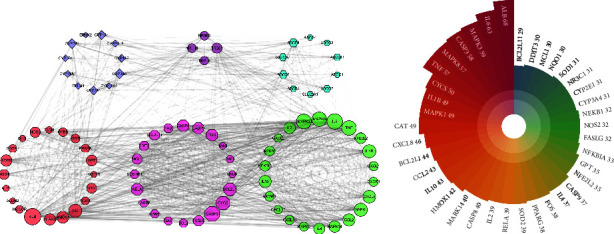
PPI analysis results. (a) Pink represents apoptosis-related targets, green represents inflammatory targets, red represents oxidative stress-related targets, light purple represents metabolic enzymes, dark purple represents nuclear receptors, and blue represents transport-related targets. (b) Critical targets with degree value greater than average.

There were 87 targets uploaded to the STRING database to obtain PPI files, and they constructed a PPI network by using Cytoscape 3.7.1 software. The PPI network contained 87 nodes and 1221 edges, with an average node degree of 28.06 (Figure 4(a)). A total of 38 targets with a degree value >28.06 were selected as key targets (Figure 4(b)). Among these, the top 10 key targets in terms of degrees of freedom were ALB, IL6, MAPK3, CASP3, TNF, MAPK8, CYCS, CAT, MAPK1, and IL1*β*, with respective degrees of freedom of 68, 63, 59, 58, 58, 57, 57, 50, 49, and 49.

### 3.6. Enrichment Analysis

**Figure 5:**
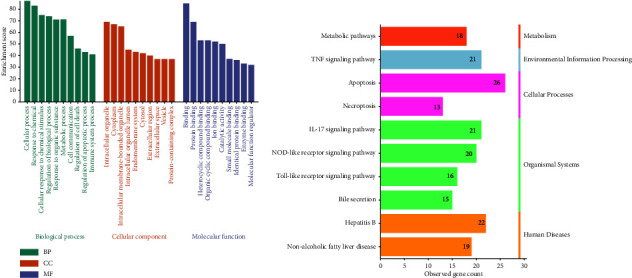
GO and KEGG pathway enrichment analyses. (a) Top 10 biological processes (BP), cellular components (CC), and molecular functions (MF). (b) Top 10 pathways of KEGG enrichment analysis from direct targets.

The GO enrichment analysis was performed to describe the gene's functions of 87 targets. GO terms include 3 categories: biological process (BP), cellular component (CC), and molecular function (MF). Among them, BP was primarily associated with the cellular process, response to chemicals, metabolic process, cell communication, regulation of cell death, and regulation of the apoptotic process. CC mainly consisted of intracellular organelle, cytoplasm, intracellular membrane-bounded organelle, and endomembrane system. MF was chiefly involved in protein binding, heterocyclic compound binding, organic cyclic compound binding, and ion binding (Figure 5(a)).

KEGG enrichment analysis showed that apoptosis, hepatitis B, IL17 signaling pathway, TNF signaling pathway, NOD-like receptor signaling pathway, nonalcoholic fatty liver disease, metabolic pathways, the toll-like receptor signaling pathway, bile secretion, and necroptosis were the top 10 signaling pathways (Figure 5(b)). The results of the KEGG analysis illustrated that the apoptosis signaling pathway, IL17 signaling pathway, TNF signaling pathway, and NOD-like receptor signaling pathway might be the main signaling pathways of KP-induced liver injury.

### 3.7. CTP Network Analysis

**Figure 6:**
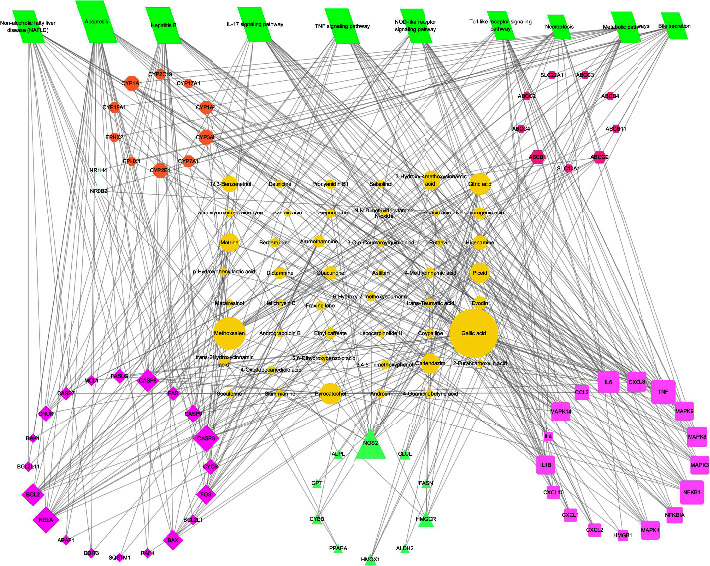
Compound-target-pathway network (yellow nodes represent compounds, green nodes represent signaling pathways, and clustered circles represent targets).

There were 10 pathways combining 47 active components and 68 targets that were used to construct a CTP network. The CTP network demonstrated 125 nodes (containing 47 chemical compound nodes, 68 target nodes, and 10 pathway nodes) and 386 edges (representing the interaction between chemical compounds, targets, and pathways) (Figure 6). According to the screening criteria, a total of 21 key targets of liver injury caused by KP were filtered (Table 2), such as IL6, TNF, IL1*β*, MAPK1, CASP3, CASP8, NFKB1, CXCL8, CCL2, CYP2E1, and CYP3A4. A total of 15 compounds had degree values greater than the mean in the analysis of topological isomerism value. According to previous research on the structure-toxicity relationship of hepatotoxic compounds [18] and literature mining [7, 19], the compounds of methoxsalen, obacunone, limonin, and dictamnine might be the material basis of the liver injury induced by KP.

### 3.8. Effects of KP on Inflammatory Factors

**Figure 7:**
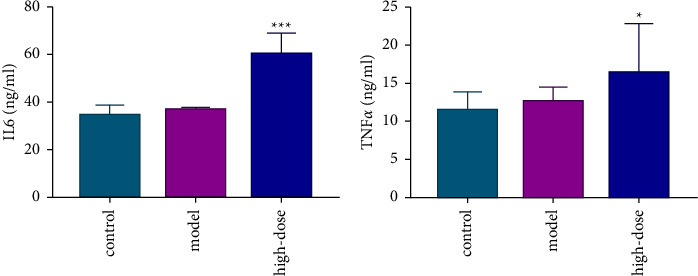
Results of ELISA experiments. (a) Changes of IL6 (*n* = 5 per group) and (b) TNF*α* levels (*n* = 5 per group) in liver tissues. Notes: ^∗^*P* value refers to the comparison between the model group and administration groups; ^∗^*P* < 0.05 and ^∗∗∗^*P* < 0.01.

Depending on the results of network pharmacology, inflammatory factors could be related to liver damage caused by KP. IL6 and TNF*α* levels in the liver were detected in control, model, and high-dose KP groups. Compared with the model group, the IL6 level in the liver was significantly increased in the high-dose of the KP group (Figure 7(a)). Compared with control and model groups, the TNF*α* level in the liver was significantly increased in the high-dose of the KP group (Figure 7(b)). The results revealed that KP caused liver damage by stimulating inflammatory factors in the inflammatory signal pathway.

### 3.9. Molecular Docking

**Figure 8:**
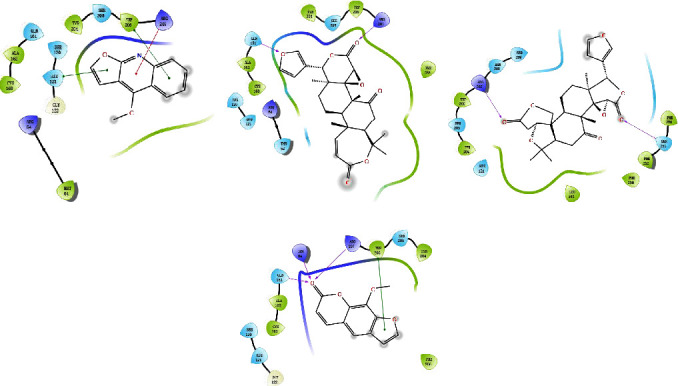
Molecular docking diagram of four compounds and CASP3. (a) CASP3-dictamnine; (b) CASP3-obacunone; (c) CASP3-limonin; (d) CASP3-methoxsalen. (Pink arrow represents hydrogen bonding, the red line represents cation-Pi interaction, and the green line represents Pi-Pi stacking).

**Figure 9:**
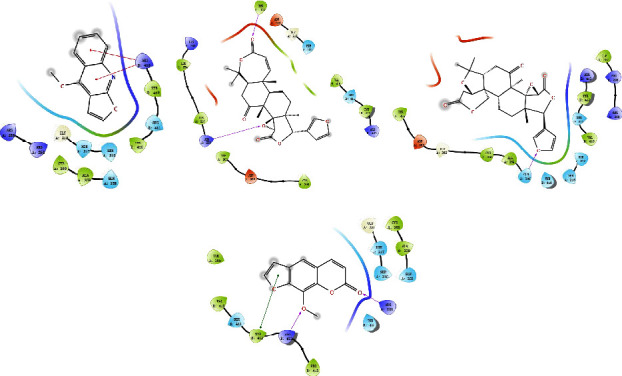
Molecular docking diagram of four compounds and CASP8. (a) CASP8-dictamnine; (b) CASP8-obacunone; (c) CASP8-limonin; (d) CASP8-methoxsalen. (Pink arrow represents hydrogen bonding, the red line represents cation-Pi interaction, and the green line represents Pi-Pi stacking).

Network pharmacology studies predicted that methoxsalen, obacunone, limonin, and dictamnine might be the main substances causing liver injury, and the mechanism may be related to apoptosis. Therefore, the molecular docking approach examined the interactions between these four major compounds and the apoptotic proteins CASP3 and CASP8. The molecular docking results demonstrated the binding affinity of methoxsalen, obacunone, limonin, and dictamnine to the targets CASP3 and CASP8 (Figures 8(a)–8(d) and 9(a)–9(d)). The results of docking scores indicated that methoxsalen had stronger binding affinity to CASP3 and CASP8 compared with the other three compounds (Table 3). Methoxsalen could form three hydrogen bonds and one Pi-Pi stacking bond with ARG207, ARG64, GLN1161, and TRP206, respectively (Figure 8(d)). Methoxsalen could form two hydrogen bonding interactions with ARG260 and ARG413 and one Pi-Pi stacking interaction with TYR412 (Figure 9(d)).

## 4. Discussion

KP has been used in the treatment of psoriasis for more than 30 years [20]. Previously, more and more concerns over its potential hepatotoxicity have been raised [11]. However, the complex composition of KP makes it difficult to investigate which component produces hepatic toxicity. Therefore, in this study, we combined the animal immune stress model, UPLC-QTOF/MS analysis, and the network pharmacology method to systematically and comprehensively study KP-induced hepatotoxicity. First, the immune stress model was used to investigate the characteristics of KP-induced liver toxicity. Second, the UPLC-QTOF/MS analysis method was applied to identify the components in KP. Third, networked pharmacologic technology was employed to rapidly screen for key compounds and mechanisms of KP-induced hepatotoxicity. The characteristics of this research method are that the combination of *in vivo* experiments and computer screening models can quickly screen hepatotoxic substances and find the main mechanisms of hepatotoxicity. The advantages of this method save the use of experimental animals, experimental costs, and time.

As the main metabolic organ and excretory organ, the liver is the main target organ for toxic injury [21]. Our findings showed that KP could cause liver cell necrosis, lobular nuclei fragmentation, and inflammatory cell infiltration in male KM mice. At the same time, there were also changes in serum ALT, AST, and AKP levels. Furthermore, the results of network pharmacology indicated that methoxsalen, obacunone, limonin, and dictamnine might be the main compounds of KP-induced hepatotoxicity. KP-induced hepatotoxicity mechanisms might be primarily through the regulation of IL6, TNF*α*, CASP3, and CASP8 targets by acting on the IL17 and apoptosis pathways.

The results of the “toxic component-target-pathway” network showed that methoxsalen, obacunone, limonin, and dictamnine might be the key compounds of KP-induced liver damage. In recent years, compounds with furans, anilines, quinones, hydrazines, thiophenes, arylpropionic acids, and alkynes in their structures may have potential hepatotoxicity [22]. Furan rings generate epoxides or cis-aldehyde after activation by metabolic enzymes that can covalently modify the biomolecules to cause cellular damage and toxicities [23]. Dictamnine is metabolized to form epoxides that conjugate with glutathione (GSH) in a very early phase. When hepatic GSH reserves become depleted, the reactive metabolites will bind to functional proteins or other nucleophiles and potentially cause necrosis [24]. Obacunone is metabolized by CYP3A4 into a cis-aldehyde intermediate, which consumes GSH to produce hepatotoxicity [25]. However, obacunone, limonin, and dictamnine in Cortex Dictamni produce hepatotoxicity that may be through additive effects. Dictamnine can not only combine with glutathione in the early stage and consume glutathione but also form adducts with liver proteins to cause liver damage. Obacunone and limonin bind mainly to lysine residues of liver proteins to produce hepatotoxicity [26]. The research shows that methoxsalen exerts hepatotoxicity by inhibiting the cytochrome CYP450 enzyme in mice [27]. These results are in accordance with network pharmacology in our study. Herein, we concluded that the material basis of hepatotoxicity induced by KP was mainly caused by the superimposed effect of furan ring compounds.

**Figure 10:**
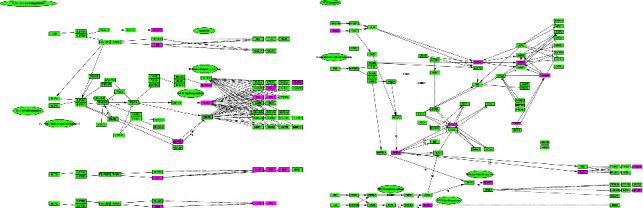
(a) IL17 signaling pathway. (b) Apoptosis signaling pathway.

At present, there are many factors and complex mechanisms of liver injury caused by TCM, including liver enzyme abnormality, immunologic reaction, oxidative stress, lipid metabolism disorder, cholestasis, hepatocyte necrosis caused by mitochondrial dysfunction, and the release of inflammatory factors [28]. During hepatocyte necrosis, the release of injury-associated molecules activates toll-like receptors on macrophages and induces proinflammatory signaling pathways leading to cytokine production. Among these proinflammatory cytokines, IL6 and TNF*α* are considered the key mediators of hepatotoxicity [29]. Our results indicated that the mechanism of KP-induced hepatotoxicity was mainly through the regulation of inflammatory targets through the IL17 signaling pathway (Figure 10(a)). ELISA experiments further indicated that KP could promote the release of inflammatory factors IL6 and TNF*α* in the liver of mice. The production of TNF*α* is closely related to the activation of Kupffer cells. Elevated TNF*α* can stimulate hepatocyte apoptosis [30]. The increased expression of TNF*α* can initiate the cleavage of pro-CASP8 and/or −CASP10 to activate CASP8/10, and CASP8/10 directly activates downstream CASP3 [31]. The results of network pharmacology indicated that KP could regulate CASP3 and CASP8 targets by acting on the apoptosis signaling pathway (Figure 10(b)). The results of molecular docking confirmed that methoxsalen, obacunone, limonin, and dictamnine had binding affinity with apoptotic proteins CASP3 and CASP8. Therefore, we speculated that the mechanism of KP-induced hepatotoxicity could be to promote hepatocyte apoptosis by promoting the release of inflammatory factors and the initiation of the apoptotic pathway.

This study also has limitations in studying the material basis and mechanisms of liver injury caused by KP. First, although the research methods of network pharmacology have developed rapidly in the past decade, the accuracy of algorithm-based prediction of related targets of compounds needs to be further improved, and the analysis methods of network topological heterogeneity results need to be more accurate. Second, the screened toxic compounds and their corresponding mechanisms need to be further verified by *in vitro* and *in vivo* experiments.

## 5. Conclusions

To conclude, we used a research method combining animal experiments, UPLC-QTOF/MS analysis, and network pharmacology to analyze toxic substances and potential mechanisms of KP hepatotoxicity. The results of animal experiments confirmed that KP was toxic to the liver of mice. The components of KP were identified by the UPLC-QTOF/MS analysis method. Through network pharmacologic analysis, methoxsalen, obacunone, limonin, and dictamnine might be the main substances of liver injury caused by KP. Its potential toxicity mechanisms might be via the IL17 signaling pathway and the apoptosis pathway acting on IL6, TNF*α*, CASP3, and CASP8 targets to exert hepatotoxic effects. Based on the results of network pharmacology, the ELISA experiments showed that KP might increase the IL6 and TNF*α* levels in liver tissues. Molecular docking results demonstrated that methoxsalen, obacunone, limonin, and dictamnine could interact with the targets CASP3 and CASP8. Our study lays the theoretical foundation for further study on hepatotoxic compounds and their mechanisms of KP.

## Figures and Tables

**Table 1:** Nonliver protective compounds of Keyin pill.

Name	Adduct	Formula	Classification of compound
(-)-Gallocatechin	M − H	C_15_H_14_O_7_	Flavonoids
(-)-Syringaresinol-4-O-beta-D-glucopyranoside	M − H	C_28_H_36_O_13_	Lignan glycosides
(S)-Salsoline	M + H	C_11_H_15_NO_2_	Tetrahydroisoquinolines
1,2,3-Benzenetriol	M − H	C_6_H_6_O_3_	Phenols
1,2,3,4-Tetrahydro-6,7-isoquinolinediol	M + H	C_9_H_11_NO_2_	Tetrahydroisoquinolines
2-Furancarboxylic acid	M − H	C_5_H_4_O_3_	Furans
2-Methyl-3-pyridinol	M + H	C_6_H_7_NO	Pyridines and derivatives
2-Phenylethyl2-O-beta-D-xylopyranosyl-beta-D-glucopyranoside	M + Na	C_19_H_28_O_10_	Organooxygen compounds
2,6-Dihydroxybenzoic acid	M + H	C_7_H_6_O_4_	Benzene and substituted derivatives
3-Hydroxy-4-methoxycinnamic acid	M − H	C_10_H_10_O_4_	Cinnamic acids and derivatives
3-Methylquinolin-4-amine	M + H	C_10_H_10_N_2_	Quinolines and derivatives
3-O-coumaroylquinic acid	M − H	C_16_H_18_O_8_	Organooxygen compounds
3-O-p-coumaroylquinic acid	M − H	C_16_H_18_O_8_	Organooxygen compounds
3,4-Dihydroxybenzaldehyde	M + H − H_2_O	C_7_H_6_O_3_	Organooxygen compounds
3,4,5-Trimethoxyphenol	M + H	C_9_H_12_O_4_	Phenols
3,5,7,3′,4′-Pentahydroxyflavanone	M + H − H_2_O, M + H	C_15_H_12_O_7_	Flavonoids
4-Guanidinobutyric acid	M + H	C_5_H_11_N_3_O_2_	Carboxylic acids and derivatives
4-Hydroxy-3-methoxyphenethyl alcohol	M + H − H_2_O	C_9_H_12_O_3_	Phenols
4-Hydroxybenzaldehyde	M − H	C_7_H_6_O_2_	Organooxygen compounds
4-Methylcinnamic acid	M + H	C_10_H_10_O_2_	Cinnamic acids and derivatives
4-O-p-coumaroylquinic acid	M + H, M + Na	C_16_H_18_O_8_	Organooxygen compounds
4-Oxododecanedioic acid	M − H	C_12_H_20_O_5_	Keto acids and derivatives
5-Hydroxy-2-(hydroxymethyl)pyridine	M + H − H_2_O	C_6_H_7_NO_2_	Pyridines and derivatives
6-Hydroxy-7-methoxycoumarin	M − H	C_10_H_8_O_4_	Coumarins and derivatives
Acutumine	M + H, M + Na	C_19_H_24_ClNO_6_	Acutumine and related alkaloids
Acutuminine	M + H, M + Na	C_19_H_24_ClNO_5_	Alkaloid
Ammothamnine	M + H	C_15_H_24_N_2_O_2_	Lupin alkaloids
Andrographidin B	M + H	C_23_H_24_O_12_	Flavonoids
Androsin	M + FA − H	C_15_H_20_O_8_	Phenolic glycosides
Apigenin 6,8-di-glucopyranoside	M − H	C_27_H_30_O_15_	Flavonoids
Astilbin	M + H − H_2_O, M + H, M + Na	C_21_H_22_O_11_	Flavonoids
Azelaic acid	M − H	C_9_H_16_O_4_	Fatty acyls
Berberrubine	M + H	C_19_H_15_NO_4_	Protoberberine alkaloids and derivatives
Carbendazim	M + H	C_9_H_9_N_3_O_2_	Benzimidazoles
Citric acid	M − H	C_6_H_8_O_7_	Carboxylic acids and derivatives
Corypalline	M + H	C_11_H_15_NO_2_	Tetrahydroisoquinolines
Dauricine	M + H	C_38_H_44_N_2_O_6_	Isoquinolines and derivatives
Dauricoside	M + H, M + Na	C_24_H_29_NO_9_	Alkaloid
Dictamnine	M + H	C_12_H_9_NO_2_	Quinolines and derivatives
Ethyl caffeate	M + FA − H	C_11_H_12_O_4_	Cinnamic acids and derivatives
Limonin	M + H − H_2_O, M + H, M + Na	C_26_H_30_O_8_	Prenol lipids
Fraxinellone	M + H − H_2_O, M + H, M + Na	C_14_H_16_O_3_	Isobenzofurans
Gallic acid	M − H	C_7_H_6_O_5_	Benzene and substituted derivatives
Helichrysin B	M − H	C_21_H_22_O_10_	Flavonoids
Higenamine	M − H	C_16_H_17_NO_3_	Alkaloid
Indole-6-carboxaldehyde	M + H	C_9_H_7_NO	Indoles and derivatives
L-Stepholidine	M + H	C_19_H_21_NO_4_	Protoberberine alkaloids and derivatives
Lecocarpinolide H	M + H − H_2_O, M + Na	C_15_H_18_O_5_	Prenol lipids
Maesopsin	M + H	C_15_H_12_O_6_	Aurone flavonoids
Matairesinol	M + H − H_2_O, M + H, M + Na	C_20_H_22_O_6_	Furanoid lignans
Matrine	M + H, M + Na	C_15_H_24_N_2_O	Lupin alkaloids
Methoxsalen	M + H	C_12_H_8_O_4_	Coumarins and derivatives
Methylsuccinic acid	M − H	C_5_H_8_O_4_	Fatty acyls
Miquelianin	M − H	C_21_H_18_O_13_	Flavonoids
Moracin M	M + H	C_14_H_10_O_4_	2-arylbenzofuran flavonoids
N, N-DimethyldecylamineN-oxide	M + H	C_12_H_27_NO	Organonitrogen compounds
Neochlorogenic acid	M − H	C_16_H_18_O_9_	Organooxygen compounds
Obacunone	M + H − H_2_O, M + H, M + Na	C_26_H_30_O_7_	Prenol lipids
p-Hydroxyphenyllactic acid	M + H − H_2_O	C_9_H_10_O_4_	Phenylpropanoic acids
Piceid	M − H	C_20_H_22_O_8_	Stilbenes
Procyanidin B1	M − H	C_30_H_26_O_12_	Flavonoids
Procyanidin C1	M − H	C_45_H_38_O_18_	Flavonoids
Pyrocatechol	M − H	C_6_H_6_O_2_	Phenols
Rutaevin	M − H, M + FA − H	C_26_H_30_O_9_	Steroids and steroid derivatives
Salsolinol	M + H	C_10_H_13_NO_2_	Tetrahydroisoquinolines
Scoulerine	M + H	C_19_H_21_NO_4_	Protoberberine alkaloids and derivatives
Sebacic acid	M − H	C_10_H_18_O_4_	Fatty acyls
Skimmianine	M + H, M + Na	C_14_H_13_NO_4_	Quinolines and derivatives
trans-2-Hydroxycinnamic acid	M − H	C_9_H_8_O_3_	Cinnamic acids and derivatives
trans-Traumatic acid	M − H	C_12_H_20_O_4_	Fatty acyls

**Table 2:** Core targets of PPI and compound-target-pathway network.

Target	PPI degree	Compound-target-pathway degree
IL6	63	11
MAPK3	59	9
CASP3	58	15
TNF	57	13
MAPK8	57	10
CYCS	50	7
MAPK1	49	9
IL1B	49	9
CXCL8	46	10
CCL2	43	6
CASP8	40	12
MAPK14	40	9
RELA	39	15
FOS	38	10
CASP9	37	8
NFKBIA	33	7
NFKB1	32	13
NOS2	32	18
FASLG	32	6
CYP2E1	31	6
CYP3A4	31	6

**Table 3:** Docking score results for compounds and targets.

Target	Compound	Docking score (kcal/mol)
CASP3	Dictamnine	−4.188
CASP3	Obacunone	−3.638
CASP3	Limonin	−3.929
CASP3	Methoxsalen	−4.968
CASP8	Dictamnine	−3.352
CASP8	Obacunone	−3.105
CASP8	Limonin	−2.754
CASP8	Methoxsalen	−4.818

## Data Availability

The data used to support the findings of this study are available from the corresponding author upon request.
